# Tooth wear and tertiary crowding: a 13-year cohort study in Amazon Indigenous populations

**DOI:** 10.1186/s40510-024-00550-2

**Published:** 2025-01-06

**Authors:** Renata Travassos da Rosa Moreira Bastos, Eduardo Oliveira da Costa, David Normando

**Affiliations:** 1 Federal University of Para, University Center of Para (CESUPA), Belém, Brazil; 2 Private Practice, Belém, Brazil; 3 Federal University of Para, Belém, Brazil

**Keywords:** Malocclusion, Tooth wear, Indigenous Population

## Abstract

**Background:**

Tooth wear is an important mechanism for reducing dental dimensions and, consequently, dental crowding. The objective of this cohort study was to examine the relation of tooth wear, adjusted for covariates (age, tooth loss, arch perimeter and intercanine width), on tertiary crowding in Amazon Indigenous populations.

**Methods:**

A sample of 40 Indigenous people in permanent dentition at T0 (baseline) and after 13 years (T1) were evaluated. The sample included 16 males and 24 females belonging to two villages, Arara (*n* = 22, mean ages 16.6 and 29.9 years) and Assurini do Xingu (*n* = 18, mean ages 16.0 and 29.6 years). Clinical, intraoral photograph and dental cast evaluations were performed at both times. The anterior crowding was measured using Little’s Irregularity Index (LI) and modeled through a multilevel linear regression with the predictor variables: village, tooth wear (T1-T0), age (T0), tooth loss (T1-T0), changes in intercanine width (T1-T0) and changes in arch perimeter (T1-T0).

**Results:**

A slight increase was observed (< 1 mm) in anterior dental crowding and a decrease in arch perimeter < 1.5 mm, while tooth wear increased between 0.65 and 0.99 units. The contextual variable (village) had no significant association with LI. In the upper arch, tooth loss was the only variable that showed an inverse association with LI (β=-0.41, *p* < 0.05). In the lower arch, the increase in dental crowding was inversely associated with tooth wear (β=-1.30, *p* < 0.05) and changes in arch perimeter (β=-0.31, *p* < 0.05). The other variables did not show significant associations.

**Conclusion:**

After 13 years, dental crowding and tooth wear increased, while the arch dimensions tended to decrease. The changes in long-term dental crowding seem to have distinct etiological components for each dental arch. In the mandible, the changes in incisor alignment were associated with increased tooth wear and decreased dental arch dimensions. Whereas in the maxilla, only tooth loss caused alterations in tooth alignment. It is suggested that the effect of increased tooth wear on the etiology of tertiary crowding is of small magnitude and restricted to the lower dental arch.

## Introduction

Dental crowding is a consequence of the discrepancy between tooth size and the dimensions of the apical base size [1, 2]. Genetic, epigenetic and environmental influences interact to produce the final phenotypes observed [3]. Some evidence indicates that crowding is a frequent characteristic in modern human populations, occurring because of increased consumption of processed and industrialized foods [1, 4–6]. Tertiary crowding is characterized by irregularities in the position of the lower incisors that occur during occlusal maturity between late adolescence and early adulthood [7]. Factors such as late mandibular growth [7], mesial migration of posterior teeth [1, 5], absence of interproximal wear [6] (common in modern human populations), and reduction in the dimensions of the dental arch along with a deficiency in the size of the alveolar bone have been related to the etiology of dental crowding [5, 6].

Tooth wear is an important mechanism for reducing the mesiodistal width of teeth and, consequently, for changes in dental crowding [1]. Begg, an Australian orthodontist, theorized in 1954 that tooth crowding was a disease of modern civilization. According to his findings, obtained from the analysis of Australian aboriginal skulls, the interproximal attrition would allow the mesial migration of teeth to their correct anatomical position. This would then reduce the severity of the malocclusion [1]. Animal study models synthesized in a systematic review performed by our research group [8], studies in human twins [9–11] and in skulls from ancestral populations [1, 12, 13], suggest mostly an environmental influence on occlusal changes observed throughout the evolution of the human species [1, 8, 10, 13].

A particular study model for this question is the assessment of the characteristics of remote populations living in relative isolation. Some Indigenous groups in the Amazon are considered semi-isolated or isolated due to the preservation of their peculiar characteristics, especially regarding their traditional eating habits [14, 15]. Among the isolated Indigenous people of the Amazon, the Yanomami had a high prevalence of dental crowding, even with severe tooth wear [14]. Studies performed by our research group among the inhabitants of the region of the Middle Valley of the Xingu River reported differences in the prevalence of dental crowding even with similar rates of tooth wear [16–18]. These data suggested that the etiology of dental crowding among Indigenous people would be predominantly associated with variations in the dimensions of dental arches, mainly related to genetic inheritance [16–19].

The study of the factors associated with tertiary dental crowding in remote Amazon populations, a sample standardized in diet and genetics, becomes more predictable when considering that many of these characteristics are not found in modern populations [16]. Longitudinal cohort studies, with a higher level of scientific evidence, are necessary to discriminate the real role of tooth wear in the etiology of dental crowding, revisiting the classic theory of Begg [1]. Tooth wear shows evidence of how human beings ate in the past [18]. Therefore, in this study, the association with the incidence of tooth wear, adjusted for covariates, on the rate of tertiary dental crowding was investigated. Two statistical hypotheses were tested to support the study. The null hypothesis (H0) defines that changes in tooth wear would not be associated with tertiary crowding. In the alternative hypothesis (H1), it was inferred that the variation in tooth wear would change the severity of tertiary crowding over time.

## Methods

### Ethical and legal statement

This study was approved by the Research Ethics Committee of the Institute of Health Sciences of the Federal University of Pará (CEP- ICS/UFPA) and by the National Ethical Committee (CONEP) for Health Sciences of Brazil (# 1.433.511). The study was also submitted for the assessment of scientific merit by the National Council for Scientific and Technological Development (CNPq). After the favorable consent of these institutions, the project was sent to the National Indian Foundation (FUNAI) to authorize researchers to enter Indigenous villages for data collection. Indigenous leaders of the Xingu were contacted prior to entering the villages.

The Indigenous population were informed of the objectives of the study and authorized their participation by signing the free and informed consent form in accordance to the Brazilian National Health Council, resolution 466/12. For the Indigenous people who did not know how to read or sign, a verbal consent was obtained through an audio recording.

### Study design, participants, and eligibility criteria

**Fig. 1:**
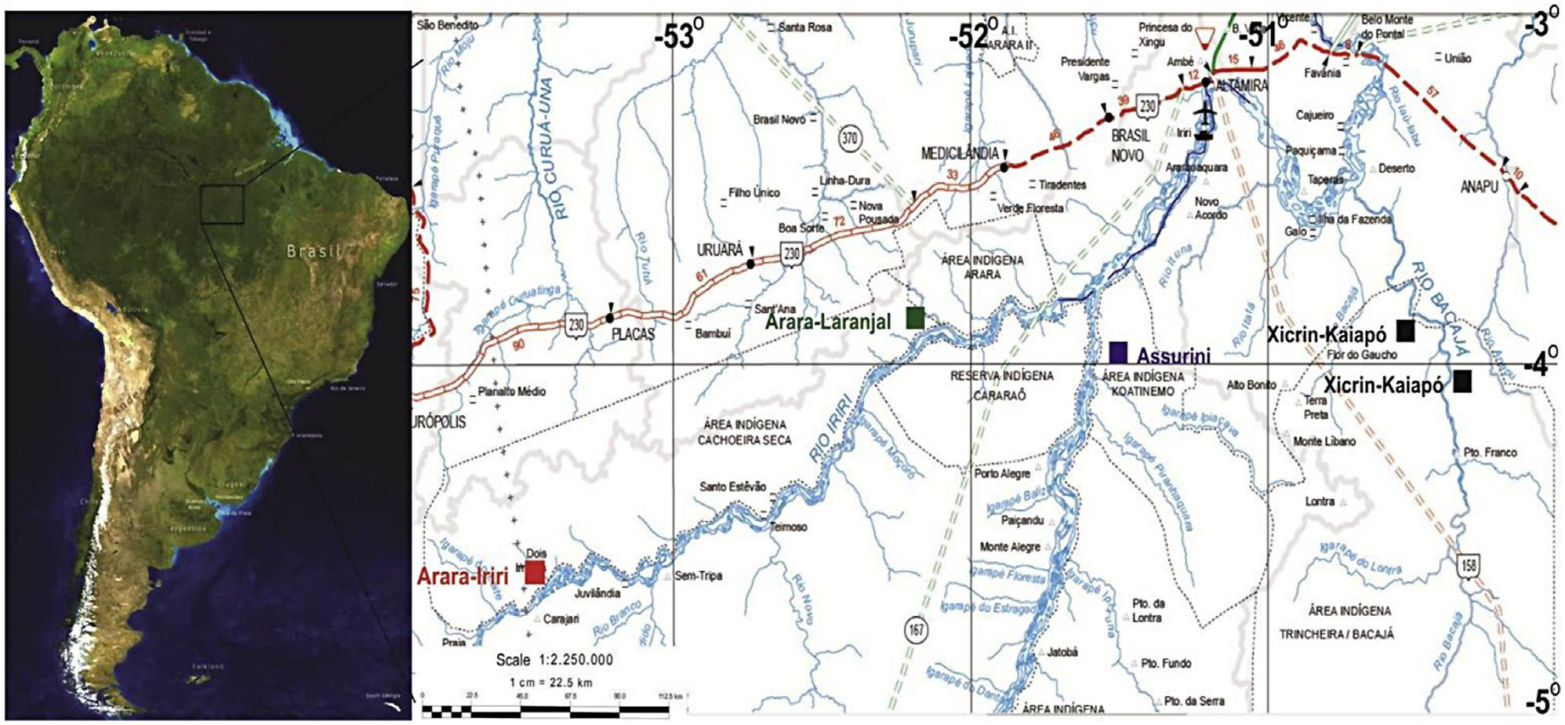
Map of the location of Arara-Laranjal (green) and Assurini do Xingu (purple) villages

This was a prospective cohort study with follow-up after 13 years, and the Strengthening the Reporting of Observational Studies in Epidemiology (STROBE) guidelines for prospective cohort studies were followed for writing the manuscript [20]. The convenience sampling was the same that had already been used in 2009 in previously published studies carried out in Indigenous populations located on the region of the Medium Valley of the Xingu River, in the villages of Arara-Laranjal (Arara do Pará ethnic group) and Koatinemo (Assurini do Xingu ethnic group), state of Pará, Brazil (Fig. 1) [16–19, 21–24].

The Indigenous people of the ethnicity Assurini do Xingu constitute an Indigenous population of Tupi nature. The first information about an official contact with this population dates back to 1971 [25]. The Arara village was first contacted in 1983 and is located 120 km from the nearest city, Altamira, in the state of Pará [18]. In the history of the Arara Indigenous people, a Karib-speaking people, there are records of several conflicts with other Indigenous groups. These conflicts were mainly with non-Indigenous people who invaded their territory, especially during the construction of the Transamazônica highway between the 1960s and 1970s. As a result, the Arara territory and its population were drastically reduced, followed by successive events of fission and fusion [26].

The eating habits of the Indigenous groups that inhabit the region of the Middle Valley of Xingu region are peculiar, predominantly traditional, and generally come from forest products based on cassava, nuts, fish, meat of wild animals, sweet potatoes, yams and fruits. The peculiar characteristics are maintained for the preparation, chewing and ingestion of food [15]. The construction of the Belo Monte Hydroelectric Dam began in 2011 and operation started in 2016, approximately 200 km away from the villages [27].

In the first expedition in 2009, a total of 66 individuals (T0) from the Laranjal, ethnicity Arara (*n* = 39), and Koatinemo villages, ethnicity Assurini (*n* = 27), were evaluated. The second trip was carried out 13 years later in November 2022 (T1) and sought to re-evaluate all previously examined individuals. The eligibility criteria considered at T0 and T1 included Indigenous people in permanent dentition under the age of 50 years old. At T0, the selected Indigenous people had all their permanent teeth. Subjects with craniofacial syndromes or anomalies, such as cleft lip and/or palate, were excluded. At T1 exclusion criteria consisted of individuals with a loss of more than eight permanent teeth, except second and third molars, or individuals with tooth loss in the anterior region of the dental arch (canines, lateral incisors or central incisors).

### Variables, data sources, and measurement

The Indigenous people selected at T0 and reevaluated at T1 were submitted to clinical, photographic and plaster model examinations to evaluate tooth wear, the biometry of the dental crowding and of the dental arch dimensions. All exams were carried out by a single researcher, an orthodontist with experience in public health and previously calibrated. An assistant researcher wrote down the characteristics observed by the researcher.

During the calibration process for tooth wear, 15% of the sample was evaluated. The inter-examiner error was analyzed using images of intraoral photographs to verify the agreement between the evaluators of the first (T0) and second (T1) expeditions. The value of the intraclass correlation coefficient (ICC) equal to 0.98 (95% CI 0.74–0.99) indicated an excellent replicability. An ICC of 0.85 (95% CI 0.64–0.94) for intra-examiner error revealed excellent replicability between clinical and photographic tooth wear assessment methods.

The clinical examination was carried out under natural light, with the aid of a flashlight (Petzl, Tikka XP2, France) and disposable tongue depressors. At T0, intraoral photographs were taken with a Canon digital camera, model EOS Rebel T3i (Tokyo, Japan) with 18-megapixel resolution. In T1, a Canon digital camera, model EOS Rebel T7 (Tokyo, Japan) with 24.1-megapixel resolution was used. Type III plaster models (Asfer, Brazil) were obtained from the upper and lower dental arches with irreversible hydrocolloid (type II alginate Jeltrate Dustless, Dentsply Sirona, USA).

The biometry of the tertiary crowding, the dependent variable, was measured using Little’s Irregularity Index (LI). The LI consists of the sum of the linear displacements of the five anatomical contact points in the region on the upper and lower incisors, verifying crowding in the horizontal plane [28].

The biometric evaluation of the dental arches was carried out by measuring the intercanine width (the greatest transverse distance between the cusp tips of the permanent canines) and the arch perimeter (the sum of the distances between the mesial points of the first permanent molars to the distal face of the canines and from the distal face of the canines to the central incisors in each hemiarch).

All biometric assessments were performed on plaster models using a digital caliper (Sylvac Fowler, Ultra-Cal Mark III, Switzerland) with 0.1 mm sensitivity. Measurements were repeated after 30 days by the same examiner. This evaluation method was used with the aim of maintaining the standardized methodology employed in the first exams at T0 [16–19, 21–24].

Tooth wear, the main independent variable, was assessed using a modification of the classification system previously described by Mockers et al. [12]. The occlusal surfaces of the second and first premolars, canines, lateral and central incisors of the upper and lower arches, were clinically examined. The following scores were recorded for each tooth: 0 = no wear; stage 1 = only enamel wear; stage 2 = dentin wear, in which the incisal/occlusal surface has more enamel than dentin; stage 3 = dentin wear, in which the incisal/occlusal surface has more dentin than enamel; or stage 4 = advanced tooth wear, near or beyond the pulp. The arithmetic mean of the recorded scores was obtained for each individual. Agreement on this characteristic was obtained in the intraoral photograph images, by the same examiner, after a period of 30 days.

Data related to sex, age and tooth loss were collected during the clinical evaluation. The difference between T1 and T0 measurements indicated changes, increase or decrease, in tooth crowding, intercanine width, arch perimeter, tooth wear and tooth loss.

### Statistical analysis

The sample was selected by convenience at both assessment times due to the size of the population, including all Indigenous people from the initial studies (T0) available to participate in T1 [16–19, 21–24], considering the exclusion criteria.

After a 30-day period, all the biometric and tooth wear variables were re-analyzed in 30% of the sample, selected randomly, with the aim of evaluating the error of the method. Systematic and random errors were verified using ICC [29] and Dahlberg’s formula [30], respectively.

Descriptive statistics by village, according to age, sex and other predictor variables were performed at T0 and T1. The normal distribution of residuals was verified by the Shapiro-Wilk test. The dependent variable, anterior dental crowding, measured by LI [28], was analyzed using a multilevel linear regression. At the first level, the following Indigenous individual characteristics were considered: tooth wear (T1-T0), age at T0, number of tooth losses (T1-T0), changes in intercanine width (T1-T0) and in arch perimeter (T1- T0), and finally, as a contextual variable, the villages. Initially, the association of each predictor variable with the outcome variable was tested in a bivariate regression model. Subsequently, only the variables that presented a statistical significance in the bivariate analysis were included in the multilevel model, considering a p-value < 0.1.

No sample size calculation was performed due to the limitation of the population size of each village. Subsequently, a *post hoc* power analysis was carried out. All statistical analyses were performed using Jamovi software (version 2.3.22, Sydney, Australia), and G*Power (version 3.1, Düsseldorf, Germany), with a significance level of 5%.

## Results

### Participants

**Fig. 2:**
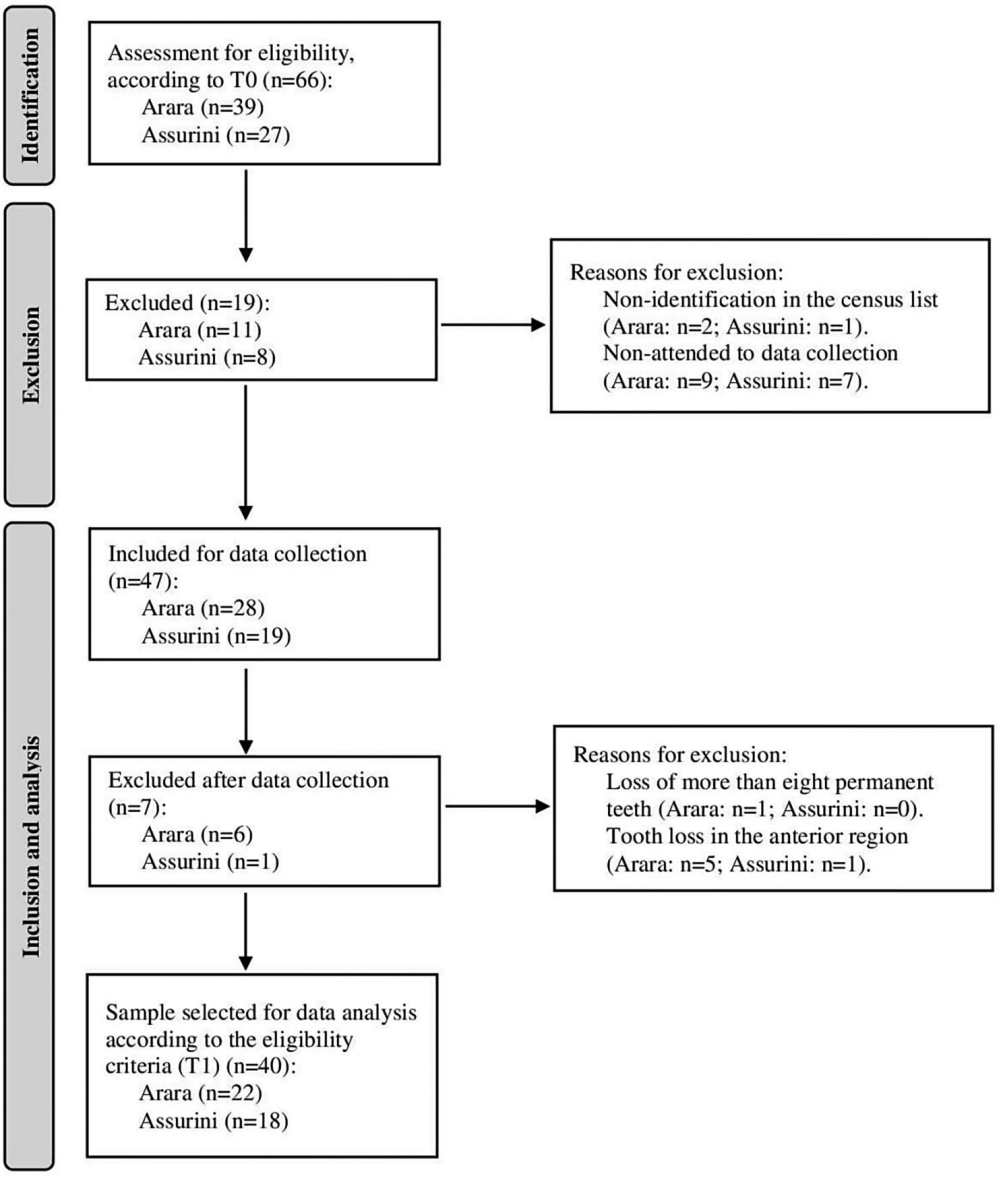
Flow diagram of selected participants from Arara-Laranjal (ethnicity Arara) and Koatinemo (ethnicity Assurini do Xingu)

In T0, according to the eligibility criteria, data were collected from 39 Indigenous people from the Arara village and 27 Indigenous people living in the Assurini do Xingu. From these total populations, 19 Indigenous people were excluded, three due to lack of identification on the census list and 16 for not attending data collection. Thus, 47 Indigenous people were examined for data collection at T1. After clinical, photographic and dental arch impression examinations, six Indigenous people from the Arara group were excluded, one because of the loss of more than eight permanent teeth and five due to tooth loss in the anterior region. For this same reason, one Indigenous person from the Assurini group was also excluded. Finally, a total of 40 Indigenous people were included for data analysis. This corresponds to a 60.6% response rate of the original sample in T0, which included 22 inhabitants from the Arara village (56.4%) and 18 from the Assurini do Xingu group (66.7%) (Fig. 2).

### Descriptive data

The final sample included a total of 40 Indigenous people, 24 females and 16 males, belonging to two groups: Arara (*n* = 22) and Assurini (*n* = 18). The follow-up time was 13 years, and the mean age at the two times was similar between the two groups, 16.0 years (± 5.83) and 16.2 years (± 5.24) at T0, and 29.6 years (± 5.91) and 29.9 years (± 5.20) in T1 in the Assurini and Arara villages, respectively (Table 1).

Dental crowding of participants from both villages varied between T0 and T1. Among the Indigenous people of the Assurini village, the mean increase was 0.79 mm (± 1.50) for the upper arch and 0.32 mm (± 1.25) for the lower arch. Data presented from the Arara-Laranjal group showed a mean increase of 0.25 mm (± 1.04) for the upper arch and 0.73 mm (± 1.32) for the lower arch (Table 1).

Occlusal/incisal tooth wear increased 0.75 index units (± 0.29) for the upper arch and 0.99 units (± 0.31) for the lower arch in the Assurini village group, and 0.65 units (± 0.26) and 0.91 units (± 0.35) for the upper and lower arches, respectively, in the Arara group (Table 1). These differences point to an approximate increase in tooth wear between 13 and 20% of the index used.

The median value for tooth losses in the upper arch in the Assurini group was 1 (0–2) and 0 (0–1) in the lower arch. In the Arara village group, the incidence of tooth loss was 0.5 (0-1.75) in the upper arch and 1 (0–1) in the lower arch. Even though the Indigenous people included in the sample at T0 had all their permanent teeth and that seven Indigenous participants were excluded after data collection due to tooth loss (Fig. 2), there was a variation in the minimum and maximum value from 0 to 3 and 0 to 2 lost teeth in the upper and lower arches, respectively, in both groups (Table 1).

For individuals from the Assurini village, the arch perimeter had a mean reduction of -0.50 mm (± 2.26), and − 0.39 mm (± 1.05), for the upper and lower arches, respectively. The intercanine width showed a mean reduction of -0.54 mm (± 0.71) in the upper arch and − 0.64 mm (± 0.62) in the lower arch. Among the Indigenous people of the Arara village, the mean reduction in arch perimeter was − 1.09 mm (± 1.41) for the upper arch, and − 1.42 mm (± 1.77) for the lower arch. For the measurement of intercanine width, the mean reduction was − 0.08 mm (± 0.88) and − 0.36 mm (± 0.71), for the upper and lower arches, respectively (Table 1).

**Table 1:** Descriptive statistics. Assessment by village (n), sex (male M/female F), mean and standard deviation (± SD) of age at T0 and T1, change of anterior crowding (LI) and of the arch perimeter, intercanine (3–3) width, tooth wear, and median and 1st and 3rd quartiles (Q1-Q3) of tooth loss, measured in the upper and lower arches

Variables		Village	
		Assurini (*n* = 18)	Laranjal (*n* = 22)
Sex (M/F)		8/10	8/14
Age T0 (± SD)		16.0 (± 5.83)	16.2 (± 5.24)
Age T1 (± SD)		29.6 (± 5.91)	29.9 (± 5.20)
LI T1-T0 (± SD)	Upper	0.79 (± 1.50)	0.25 (± 1.04)
	Lower	0.32 (± 1.25)	0.73 (± 1.32)
Tooth wear T1-T0 (± SD)	Upper	0.75 (± 0.29)	0.65 (± 0.26)
	Lower	0.99 (± 0.31)	0.91 (± 0.35)
Tooth loss T1-T0 (Q1-Q3)	Upper	1 (0–2)	0.5 (0-1.75)
	Lower	0 (0–1)	1 (0–1)
Arch perimeter T1-T0 (± SD)	Upper	-0.50 (± 2.26)	-1.09 (± 1.41)
	Lower	-0.39 (± 1.05)	-1.42 (± 1.77)
3–3 width T1-T0 (± SD)	Upper	-0.54 (± 0.71)	-0.08 (± 0.88)
	Lower	-0.64 (± 0.62)	-0.36 (± 0.71)

Regarding the systematic error, an ICC of 0.73 (0.32–0.91) was observed between the two measurements of tooth wear for the upper arch and 0.93 (0.79–0.98) for the lower arch, indicating moderate to excellent replicability. A small random error for each arch was observed, 0.12 units for the upper arch and 0.08 units for tooth wear in the lower arch. The systematic error for LI revealed an ICC of 0.89 (0.66–0.96) and 0.91 (0.74–0.97) for the upper and lower arches, respectively, indicating excellent intra-examiner replicability. A random error of 0.28 mm for the upper arch and 0.24 mm for the lower LI was verified. Finally, an ICC that ranged from 0.88 (0.66–0.96) for the variable lower perimeter to 0.97 (0.89–0.99) for the upper intercanine width demonstrated, again, excellent replicability. The Dahlberg value ranged from 0.15 mm for the upper intercanine width to 0.56 mm for the upper perimeter, which denotes the precision of the method and satisfactory calibration of the examiner (Table 2).

**Table 2:** Study error analysis. Systematic error (ICC), 95% confidence interval (CI) and random error (Dahlberg’s formula) for the variables dental crowding (LI), tooth wear, intercanine (3–3) width and arch perimeter

Variable	Systematic error ICC (CI 95%)	Random error (Dahlberg)	
*Maxilla*			
LI	0.89 (0.66–0.96)	0.28	
Tooth wear	0.73 (0.32–0.91)	0.12	
3–3 width	0.97 (0.89–0.99)	0.15	
Arch perimeter	0.92 (0.74–0.97)	0.56	
*Mandible*			
LI	0.91 (0.74–0.97)	0.24	
Tooth wear	0.93 (0.79–0.98)	0.08	
3–3 width	0.89 (0.67–0.97)	0.21	
Arch perimeter	0.88 (0.66–0.96)	0.36	

### Main results (regression analysis)

#### Upper arch

**Fig. 3:**
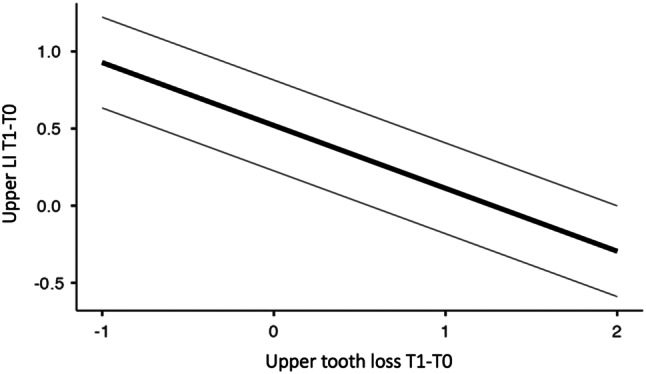
Line plot representing upper dental crowding modeled by the number of tooth losses

The ICC value, which was the indicator of the degree of dependence within the cluster, equal to approximately 0.16, means that 16% of the variation in the upper anterior dental crowding occurs between the village groups. Therefore, the village groups could be evaluated together, within the same cluster, as the correlation value was low. Corroborating this fact, the p value of the likelihood test (LRT) showed that there was no influence of the village assessed at an isolated level on the upper dental crowding (*p* = 0.167) (Table 3; Fig. 3).

The upper and lower arches were analyzed separately to maintain the assumption of the independence of the Y values (dependent variable) of the regression model. For the upper arch, the evaluation of the predictor variables based on the bivariate regression model revealed that the changes in tooth wear between T0 and T1 (*p* = 0.016) and the number of tooth losses in the same period (*p* = 0.004) presented a statistically significant association with the changes in the maxillary anterior dental crowding (LI). The other variables did not reach a value of *p* < 0.1. The multicollinearity assumption showed a variance inflation factor value lower than five, indicating the absence of collinearity between the two predictor variables under investigation. The assumption of homoscedasticity of the residuals was also fulfilled, visualized through a graphical analysis of the residuals. Finally, there was normality in the residual distribution of upper tooth wear (T1-T0) and the number of upper losses (T1-T0) verified by the Shapiro-Wilk test (*p* = 0.262).

Once all assumptions were met for the upper arch, the multilevel linear regression analysis was performed (Table 3). The adjusted R^2^ value equal to 0.206 indicates that, together, the upper tooth wear (T1-T0) and the number of upper tooth losses (T1-T0) can explain 20.6% of the variation in the upper anterior dental crowding between T0 and T1.

**Fig. 4:**
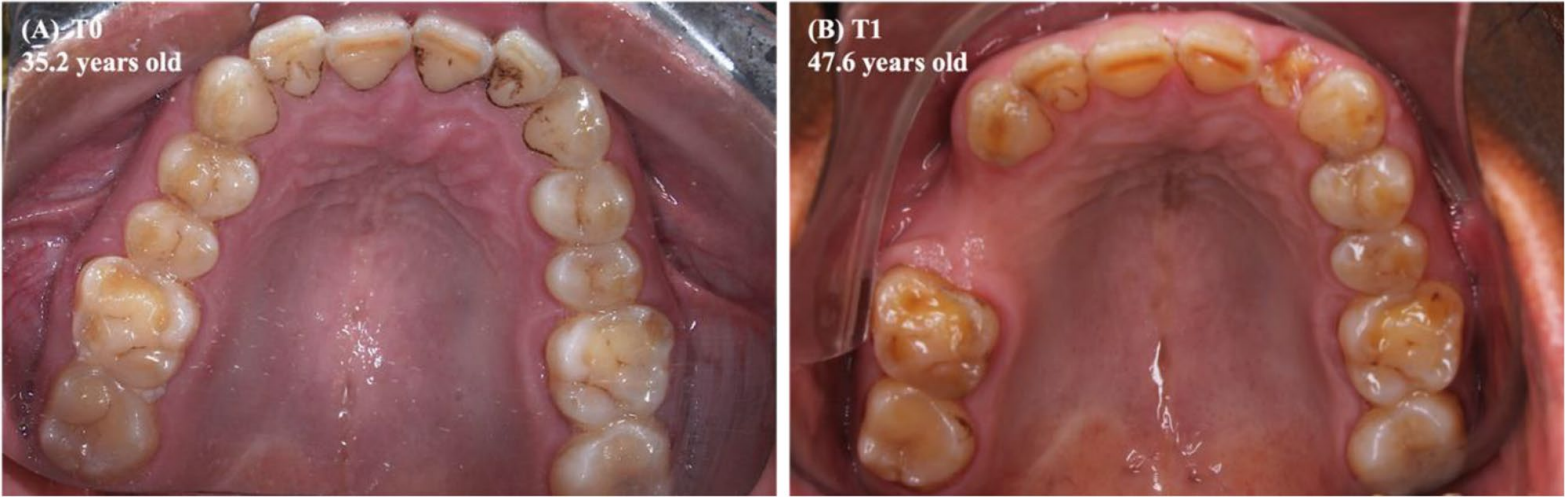
Intraoral photographs of the maxillary arch of a male Indigenous of Arara ethnicity. Legend: Absence of tooth loss in T0 **(A)** and loss of 3 permanent teeth in T1 **(B)** (1.5, 1.4, 2.2). The difference in the LI (T1-T0) was equal to -2.28 mm, demonstrating a reduction in upper anterior crowding in association with an increase in the number of tooth losses

Among the predictor variables that presented a statistically significant result in the bivariate analysis and that were included in the multilevel linear model, only the number of upper tooth losses (T1-T0) (*p* = 0.021) maintained a statistically significant and inverse association with the upper anterior dental crowding (Table 3; Figs. 3 and 4). When interpreting the beta value (β, estimate), it was noted that for each upper tooth loss, there was a reduction of 0.4 mm in the upper anterior dental crowding after 13 years.

**Table 3:** Bivariate and multilevel linear regression model (level 1 individuals, level 2 village groups) of the upper arch for the association between the predictor variables and the increase in upper anterior dental crowding (dependent variable). Adjusted R^2^ = 0.206

Independent variables	Bivariate model			Multilevel linear model						Sample power
	*p*-value	CI (95%)		*p*-value	β	CI (95%)		ICC	*p*-value (LRT)	
		Lower	Upper			Lower	Upper			
(Village)								0.158	0.167	0.796 (79.6%)
*Maxilla*										
Tooth wear T1-T0	0.016*	-3.181	-0.343	0.064	-1.326	-2.687	0.0349			
Tooth loss T1-T0	0.004*	-0.846	-0.169	0.021**	-0.408	-0.740	-0.0755			
Age T0	0.693	-0.0916	0.0616							
Arch perimeter T1-T0	0.149	-0.0604	0.382							
3–3 width T1-T0	0.916	-0.5303	0.478							

CI: confidence interval; *Statistical significance at *p* < 0.1; ** Statistical significance at *p* < 0.05; ICC: intraclass correlation coefficient; LRT: likelihood test; 3–3: intercanine

#### Lower arch

**Fig. 5:**
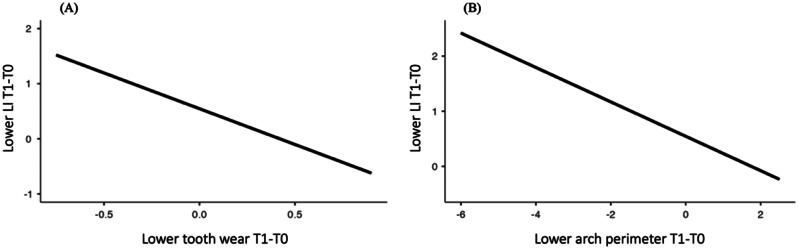
Line plots representing lower dental crowding modeled by **(A)** lower tooth wear and **(B)** changes in the lower arch perimeter

The value of the indicator of the degree of dependence within the cluster equal to 0.00 indicated there was no correlation of the variation in the lower anterior dental crowding between the village groups. Confirming this data, the p value of the likelihood test shows that there was no influence of the village assessed at a separate level on lower dental crowding (*p* = 1.000) (Table 4; Fig. 5).

For the lower arch, the evaluation of the predictor variables based on the bivariate regression model showed that the arch perimeter (T1-T0) (*p* = 0.093), the intercanine width (T1-T0) (*p* = 0.075) and the age at T0 (*p* = 0.082) presented a statistically significant association with the changes in the lower anterior dental crowding (LI). The other exploratory variables did not reach a value of *p* < 0.1. The multicollinearity assumption showed a value of the variance inflation factor lower than five, and there was also compliance with the assumption of homoscedasticity, visualized through a graphical analysis of the residuals. Finally, there was normality in the residual distribution verified by the Shapiro-Wilk test (*p* = 0.223).

Once all assumptions were met for the lower arch, the multilevel linear regression analysis was performed (Table 4). For this model, age at T0 was not included and the predictor variable of interest in the study, tooth wear (T1-T0), was added. Based on the adjusted R^2^ value equal to 0.173, it can be inferred that 17.3% of the variation in the lower anterior crowding can be determined by lower tooth wear (T1-T0), by changes in the lower arch perimeter (T1-T0) and by the lower intercanine width (T1-T0).

**Fig. 6:**
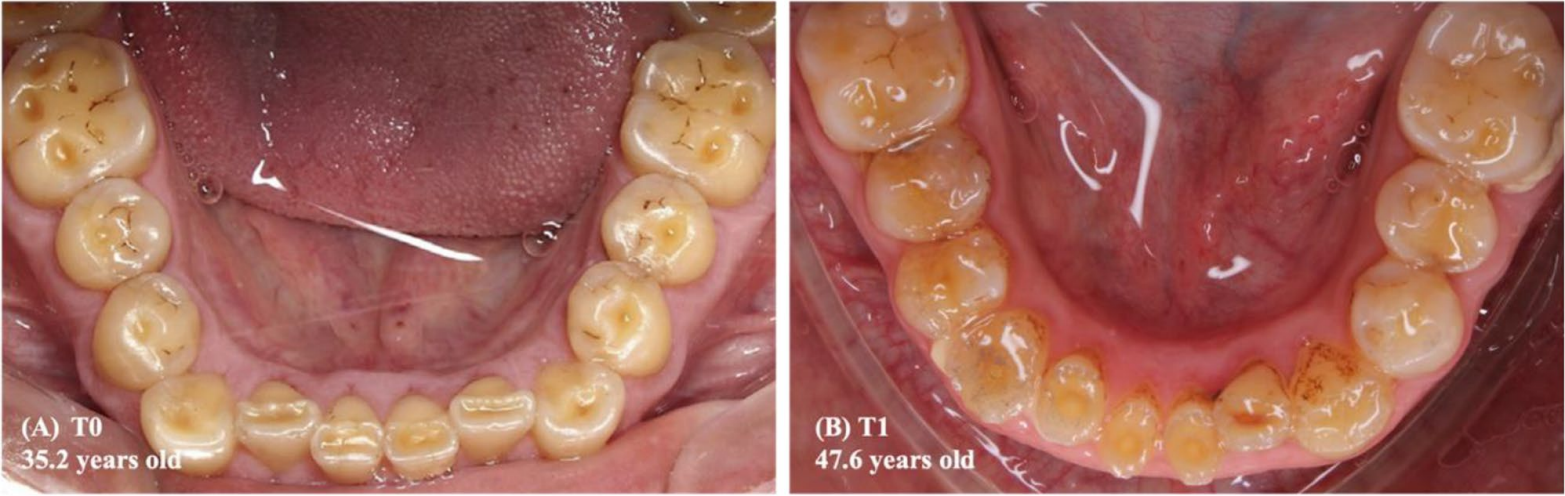
Intraoral photographs of the mandibular arch of the same Indigenous person described in Fig. 4. Legend: In 2009 **(A)** and 2022 **(B)**, the increase in the arithmetic mean of tooth wear scores (T1-T0) was equal to 1.10. The difference in the arch perimeter (T1-T0) was equal to 0.55 mm. In turn, the difference in the LI (T1-T0) was equal to -2.85 mm, which suggests a reduction in lower anterior crowding in association with increased tooth wear

**Fig. 7:**
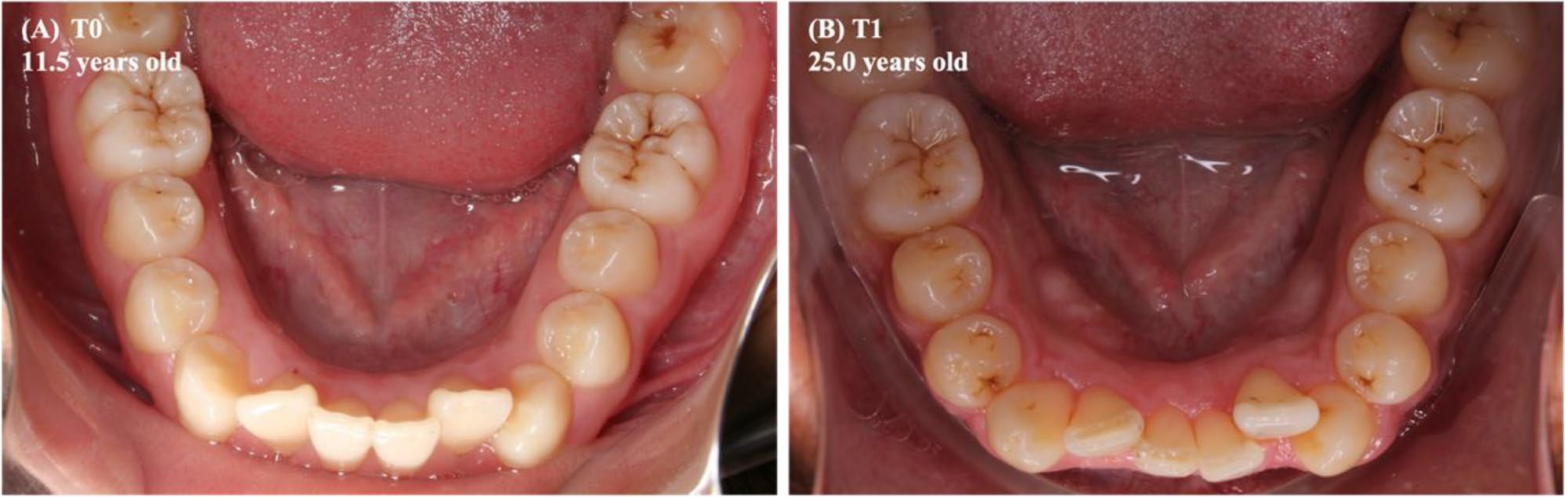
Intraoral photographs of the mandibular arch of a male Indigenous person of Arara ethnicity, in 2009 **(A)** and 2022 **(B)**. Legend: The increase in the arithmetic mean of tooth wear scores (T1-T0) was equal to 0.90. The difference in the arch perimeter (T1-T0) was equal to -3.16 mm. In turn, the difference in the LI (T1-T0) was equal to 5.55 mm, which suggests a greater increase in lower anterior crowding in association with a greater reduction in the arch perimeter

Among the predictor variables taken into the multilevel linear model, the lower tooth wear (T1-T0) (*p* = 0.043) and the changes in the lower arch perimeter (T1-T0) (*p* = 0.022) exhibited a statistically significant and inverse association with the lower anterior dental crowding (Table 4; Figs. 5, 6 and 7). The beta value (β, estimate) revealed that in each increase in the mean score of the lower tooth wear, the increment in the lower anterior dental crowding was smaller, approximately 1.3 mm. Furthermore, the greater reduction in the lower dental arch perimeter, in millimeters, led to a 0.3 mm increase in lower anterior crowding (T1-T0).

**Table 4:** Bivariate and multilevel linear regression model (level 1 individuals, level 2 village groups) of the lower arch for the association between the predictor variables and the increase in lower anterior dental crowding (dependent variable). Adjusted R^2^ = 0.173

Independent variables	Bivariate model			Multilevel linear model						Sample power
	*p*-value	CI (95%)		*p*-value	β	CI (95%)		ICC	*p*-value (LRT)	
		Lower	Upper			Lower	Upper			
(Village)								0.00	1.000	0.623 (62.3%)
*Mandible*										
Tooth wear T1-T0	0.292	-1.9305	0.597	0.043**	-1.298	-2.508	-0.0873			
Arch perimeter T1-T0	0.093*	-0.483	0.0390	0.022**	-0.312	-0.568	-0.0562			
3–3 width T1-T0	0.075*	-1.139	0.0573	0.068	-0.521	-1.065	0.0225			
Age T0	0.082*	-0.140	0.00882							
Tooth loss T1-T0	0.231	-0.989	0.246							

CI: confidence interval; *Statistical significance at *p* < 0.1; ** Statistical significance at *p* < 0.05; ICC: intraclass correlation coefficient; LRT: likelihood test; 3–3: intercanine

A *post hoc* power analysis was performed based on a multiple linear regression model. For the upper arch, the power of the test was equal to 79.6%, considering the R^2^ value equal to 0.206 to calculate the effect size, the α error equal to 0.05, the sample size equal to 40 modulated for two predictor variables (Table 3). Finally, for the lower arch, the power of the test was 62.3% considering the R^2^ equal to 0.173 to calculate the effect size, the α error equal to 0.05, and the sample size equal a 40 modulated for three predictors (Table 4).

## Discussion

The genetic diversity of small and semi-isolated populations in the Amazon, even those with common characteristics and genetic origin, depends on the interaction of factors such as the genetic composition of the ancestors, the demographic history of the population, the degree of isolation, genetic flow, and the selective effect of environmental factors on individuals [26]. Some traditional people of the Amazon, inhabitants of the Middle Valley of the Xingu, are subject to similar environmental conditions which is confirmed by similar eating habits and the same pattern of tooth wear between groups of different ethnicities [16–18, 23]. Additionally, studies in human genetics point to a large intergroup genetic distance and a small intragroup diversity of the population evaluated in the present study [26, 31, 32]. This is confirmed by the discrimination of semi-isolated Indigenous groups in the Amazon with high accuracy in epidemiological studies evaluating the dentofacial biometry of these individuals [16, 21, 22]. Thus, these populations constituted a unique opportunity to analyze the role of genetics and the environment in determining malocclusions, especially dental crowding.

The impact of genetics [14, 17, 18] and the environment [1, 8, 11, 13] on the etiology of dental crowding has been evaluated through cross-sectional studies, which characterizes a low level of evidence. As this topic is still highlighted in scientific literature, this cohort is justified. This is an unprecedented longitudinal study with 13 years of follow-up of two Indigenous groups of different ethnicities. The assessment of the occlusal/incisal tooth wear was performed using a descriptive method [12] whose reliability in clinical and intraoral photograph assessments has already been previously tested and validated [33]. The use of biometric measurements of the dental arches and of the dental crowding index in plaster models was the quantitative method chosen to maintain a standardized methodology already used in previously published studies [16–19, 21–24]. Therefore, solid parameters were reached to allow the comparison of results obtained at T0 and T1. The results of the systematic measurement errors of tooth wear and anterior crowding in the upper and lower arches, which indicated moderate to excellent replicability, demonstrated satisfactory calibration by the evaluator before carrying out the study. The random error of less than 0.3 mm showed a good accuracy of the methods used for quantitative and qualitative data analyses (Table 2).

Variations of tooth wear measurements, of biometry of the maxilla and mandible, and of the incidence of tooth loss over the 13 years of follow-up of this cohort suggested an impact of these covariates on LI in the Indigenous people evaluated.

In the maxilla of the Assurini and Arara Indigenous groups, a reduction in the mean of upper dental crowding was associated with an increase in tooth loss. This suggested an inverse and statistically significant relationship (Table 3; Figs. 3 and 4).

Tooth loss represents an important clinical marker of oral health. It is an early indicator of the health-disease process and social inequality, especially in vulnerable groups such as Indigenous people [34–36]. Corroborating these findings in a longitudinal analysis of urban populations [37], the risk of tooth loss was associated with the baseline data related to the tooth status (previous tooth loss, periodontal condition and tooth decay) and sociodemographic variables, such as age, smoking and education level. Regarding the last variable, a higher level of education was associated with a lower risk of tooth loss, and education and social inequality are determinants that go side by side in an inversely proportional relationship.

The isolation of the traditional people of the Amazon and the consequent absence of urban commerce makes the environment conducive to a healthier diet. This is characterized by the consumption of fish and natural products, and less contact with processed and industrialized foods, ultimately contributing to better oral health conditions [38]. On the other hand, the history of recent contact with urban populations has changed the dynamics of the health-disease process of these populations. Even with access to general health and dental services, the focus is on curative assistance for diseases instead of health promotion and disease prevention. Therefore, tooth loss, which is the endpoint of oral disease, occurs more often [39]. In a comparison of the magnitude of inequalities in oral health between Indigenous and non-Indigenous populations, the original people had worse oral hygiene conditions, a greater amount of tooth loss, untreated tooth decay and severe periodontal disease [40, 41].

In this context, the construction and inauguration in 2019 of the Belo Monte Hydroelectric Dam, on the Xingu River, brought a series of environmental, cultural, social and economic impacts to Indigenous people who lived there [42]. The approach to urban activities and commerce occurred in two main ways: through the arrival of thousands of people in the region, attracted mainly by employment opportunities [27], and through the displacement of at least 20,000 individuals from that region. The displaced individuals included Indigenous, riverine and quilombola people, which led to disastrous consequences in terms of maintaining the tradition of their customs [43–46]. All these social and economic impacts probably contributed to the change in eating patterns observed in recent years [1, 6, 47, 48], as well as the arrival of processed sugar-based foods, provided by visitors [42]. A soft diet rich in fermentable carbohydrates, especially sugar, directly impacts the occurrence of tooth decay and consequently of tooth loss [47–49]. Furthermore, an absence of 16 Indigenous people during the data collection period, exemplifies the continuous process of fission in increasingly smaller groups of Indigenous populations of the Xingu [43, 44].

Data related to the health of Indigenous populations in Brazil revealed that tooth loss, tooth decay, periodontal disease, tooth wear and malocclusion are the main oral health problems faced by Amazon people of different ethnicities [50, 51]. The high prevalence of caries and malocclusion among Indigenous people suggests, concomitantly, the inequalities in social and oral health to which this people are subject in relation to other populations [52]. However, data from Indigenous people related to tooth loss and especially its association with dental crowding, are still scarce in the scientific literature. Similarly, studies that evaluate the effect of tooth loss on arch alignment among non-Indigenous individuals are also meager.

Thus, corroborating the results of the present study, the evaluation of occlusal changes in an urban population with normal occlusion during 47 years of follow-up demonstrated that individuals without tooth loss showed a significant worsening in dental alignment when compared to individuals with tooth loss [53]. Certainly, the tooth loss observed among Indigenous people corroborates to a lesser worsening of tertiary crowding over time.

In the mandible, the smallest increase in dental crowding was associated with increased tooth wear. Still, inversely, and statistically significant, the lower anterior crowding increased the greater the reduction in the perimeter of this arch (Table 4; Figs. 5, 6 and 7).

Data from a systematic review revealed that dietary consistency in animals seems to interfere with dental and dental arch changes. An increased chewing load was associated with larger dental arch dimensions and a more severe pattern of tooth wear, especially on the occlusal surface [8]. Furthermore, when demonstrating the role of environmental changes related to a lower food consistency with the evolution of the human species, it is noted that increasingly smaller dental arches and wider teeth, which in turn are less worn, are associated with a negative tooth-bone discrepancy [1, 6].

Tooth wear is a physiological consequence of occlusion aging [14, 23, 54–57]. Although interproximal wear is directly related to the magnitude and frequency of occlusal forces, occlusal wear rates also depend on the physical properties of ingested food [58]. Even considering the controversial results in the literature, studies demonstrated a positive association between occlusal and interproximal tooth wear visualized through facet areas [58, 59]. This is likely to justify the improvement in anterior alignment over the years in this cohort. Moreover, evidence indicated the average maximum bite force tends to be greater among Indigenous people than in urban populations. This is closely related to dietary habits that provide greater stimulus of the masticatory system in these people [60]. Therefore, it is expected that it justifies the strong relationship between tooth wear with dietary patterns and chronological age of remote Indigenous populations in the Amazon. However, the same cannot be said for a control group constituted by an urban population [23].

Anthropological-based studies involving ancestral populations point to contradictory results regarding the etiology of dental crowding. The assessment of fossils excavated from ancient Egyptians revealed the presence of more aligned teeth, in association with a large amount of tooth wear on the occlusal surfaces [2]. Similarly, Begg argued that the relatively low incidence of malocclusion in Stone Age men occurred largely due to the reduction in tooth size, which was partly observed from the results of this cohort for the lower arch [1]. On the other hand, an increased rate of dental crowding was observed in the evaluation of Indigenous people [61], even those with pronounced tooth wear [14].

New findings minimize the widespread influence of tooth wear on occlusal variation of human populations, which is direct evidence of what an individual ate in the past [18]. It is noted that dental crowding in the permanent dentition, especially among semi-isolated traditional people, is present and even associated with a large amount of tooth wear [14]. For this reason, cross-sectional epidemiological studies carried out among inhabitants of the Xingu River suggested a genetic determinism in the etiology of malocclusion. This genetic determinism is characterized by variations in the dental arch dimensions and their effect on dental crowding, which is independent of the chewing activity that this population is subjected to [16–19]. It is also worth highlighting that in individuals with normal occlusion evaluated over a long period, changes in the size and alignment were observed. In the same way that there was a decrease in the mesiodistal size of the teeth, there was also a worsening of crowding in the incisor region [62]. The reduction in the dimensions of dental arches over the years was probably largely responsible for the worsening of anterior alignment and not tooth wear, confirming the genetic preponderance of this malocclusion.

Several longitudinal studies that evaluated occlusal changes in individuals who have not undergone orthodontic treatment are unanimous in finding that there was an increase in the rate of dental crowding, especially affecting the anterior region of the dental arches [63–65]. These data may be related to the transverse movement lingual tipping of the canines and lower premolars [66, 67] and reducing the length of the mandibular arch [68]. Similarly, the literature showed that from adolescence to late adulthood, in a longitudinal follow-up of 40 years, there was an increase in anterior crowding in the mandible. Dimensional changes in dental arches, such as reduction in intercanine distance, arch perimeter and length, among other changes, probably explain the anterior crowding of these individuals [62]. In some cases, space loss was greater in the mandible than in the maxilla, without being a sexual dimorphism in this condition [63, 68].

Finally, according to the variables considered in this cohort, there was no need to evaluate the two villages into separate clusters for both upper and lower arches. This suggests that even considering a large genetic distance between groups and a small intragroup genetic diversity [26, 31, 32], the Arara and Assurini Indigenous groups experience living in the same environmental conditions [15], which was imperative for the changes in tooth wear and the biometry of dental arches and crowding observed.

From an orthodontic perspective, the inherited genetic factors related to the size of the alveolar bone, namely arch morphology (arch perimeter, intermolar and intercanine width) [9, 10], and tooth size [11] are dominant in the etiology of the tertiary crowding [10]. However, the anthropological view related to the transition from the traditional to the modern dietary pattern cannot be disregarded. As an individual’s chewing function transitions from the traditional to the modern diet, they need to adapt to changes in their chewing function. This adaptation seems to be related to the deficiency in the growth and development of the alveolar bone. This suggests the environmental factor as an etiological agent, even if secondary, on crowding and dental malocclusion [2, 8]. Face and occlusion must be understood as a morphogenetic heritage that is potentially predetermined and subjected to external influences [69]. Future research that investigates dental crowding and considers the importance of genetics and environment on its determination, should be planned. The research should identify the extent to which biometric variables of the dental arches, not analyzed in this cohort, impact the frequency and severity of this condition in different populations. While scientific evidence has shown that anterior dental alignment worsens over time, orthodontists plan and control anterior dental alignment with retainers.

### Limitations

Among the main limitations of this study, the high incidence of tooth loss in the sample after a period of 13 years of follow-up (T0 to T1) stood out. Only 35% of the sample under analysis (*n* = 14/40) did not present tooth loss at T1. Still, 65% of Indigenous people lost at least one tooth. Therefore, tooth loss, one of the predictor variables, may have influenced the reduction of dental crowding.

The difficulty in locating three Indigenous people on the census list in T1 and the absence of 16 participants from the initial sample (*n* = 16/66) reduced the study sample, which was already considered limited, by 28.8% (*n* = 19/66). It is likely that this sample loss was related to the construction of the Belo Monte Hydroelectric Dam and the resulting proximity to the urban environment. These factors contributed greatly to the progressive change in the semi-isolation dynamics of the villages, as there was the displacement of many Indigenous and other traditional people, as well as the division of Indigenous populations in the Xingu region into smaller population groups [43, 44].

The biometric assessment of dental crowding in incisors and wear on the incisal surface may have generated a smaller increase in LI. This is because the greater the incisal wear, the smaller the linear distance between the anatomical point of contact in the region of the upper and lower incisors.

Finally, the sample of this study was considered limited. Although the *post hoc* power analysis for the lower arch reported a value less than the 80% of the reference used for studies in the health area, there was no impact on the result of the multilevel linear regression model. A small effect of less than 20% was statistically detected even with a limited sample, which eliminates the risk of a false negative.

Future prospective studies should be designed with the intent to analyze the impact of other biometric measurements of dental arches on dental crowding, as well as to analyze the facial changes to which these traditional Amazon people are subjected to over time. Thus, new evidence regarding the etiology of malocclusions can be suggested, especially dental crowding, in Indigenous people from the Amazon.

## Conclusion

A different impact on the tertiary crowding in both arches was not observed when comparing the two villages. On the other hand, dental arches had different behaviors regarding the variables that explain changes in crowding over time. For the lower arch, worsening in incisor alignment was associated with a smaller increase in tooth wear and a greater reduction in the dental arch perimeter. However, in the maxilla, these predictor variables were not associated, and only tooth loss was able to lead to an improvement in tooth alignment. In addition to the role of genetics, a secondary influence of tooth wear on the etiology of tertiary crowding in the lower arch is suggested, which partially ratifies Begg’s theory.

## Data Availability

The data presented in this study are available upon reasonable request from the corresponding author.
